# Assessing Functional Status in Geriatric Patients Attending Primary Healthcare Centers in Qassim Region, Saudi Arabia

**DOI:** 10.7759/cureus.62012

**Published:** 2024-06-09

**Authors:** Amirah Abumismar, Saulat Jahan

**Affiliations:** 1 Family Medicine, Qassim Health Cluster, Buraidah, SAU

**Keywords:** primary healthcare, saudi arabia, geriatric, functional status, activities of daily living, ablution

## Abstract

Background

There is an increase in the geriatric population globally. Also, in Saudi Arabia, the elderly population is expected to become a significant proportion of the total population in future decades. To provide comprehensive care to the geriatric population, an assessment of their functional capacity is crucial.

Objectives

This study aims to assess functional capacity and identify factors associated with functional impairment among geriatric patients at primary healthcare centers (PHCCs) in Qassim Region, Saudi Arabia.

Methods

A cross-sectional study was conducted among 310 geriatric patients, including 155 males and 155 females, attending PHCCs. An interviewer-administered survey was conducted from June through October 2023. The interviews were conducted by trained physicians. To assess functional capacity, the Katz index of independence in activities of daily living (Katz ADL), the Lawton-Brody instrumental activities of daily living (Lawton-Brody IADL) scale, and Wudu (ablution) performance were used as assessment tools. The data was collected via a Google Form (Google LLC, California, USA) through an interviewer-administered questionnaire. The data was analyzed using SPSS Statistics version 21 (IBM Corp. Released 2012. IBM SPSS Statistics for Windows, Version 21.0. Armonk, NY: IBM Corp.).

Results

The mean age of the study participants was 71.9 (±7.02) years. A vast majority (91.9%) had chronic diseases. Among basic ADL, the respondents had a high level of independence in feeding (99.4%) and transferring (95.5%), while there was a low level of independence in bathing (13.2%). The analysis of the Lawton-Brody IADL showed independence in medication management (75.8%) and telephone use (72.9%); however, 54.8% of the study participants were unable to perform laundry-related activities independently. Around three-fourths (76.8%) of the study participants were able to perform Wudu independently. IADL independence was statistically significantly associated (p<0.001) with age, gender, education, and chronic diseases. The Katz ADL and Lawton-Brody IADL were correlated (r=0.607, p<0.0001), and Wudu performance was positively correlated with both indices (r=0.636, r=0.60, p<0.0001).

Conclusions

Assessing elderly functional capacity and addressing the risk factors of functional impairment is crucial to improving the quality of life in this segment of the population. Future research is needed to validate the use of Wudu performance as an assessment tool for functional capacity in the elderly population.

## Introduction

The elderly population is progressively increasing worldwide. It is estimated that one in six people will be 60 years of age or older by 2030, and by 2050, the global population of the elderly will double to approximately 2 billion [1]. In Saudi Arabia, according to the 2017 General Authority of Statistics elderly survey, 4.19% of the total Saudi population comprises the elderly population aged 65 years and older [2]. It is predicted that the elderly population will be about 25%, which is around 10 million out of 40 million of the total Saudi population by 2050 [3].

The gradual increase in the elderly population calls for health and medical service improvements and enhancement of geriatric care provision and chronic illness prevention for an improved quality of life for this segment of the population. However, several factors influence the health and functional status of geriatricians. These factors include chronic illnesses, physiological decline, psychological and cognitive decline, and other acquired medical conditions [4,5].

Functional status is the ability to perform basic activities of daily living (BADL) and complex ADL, reflecting independence and quality of life. Functional status is a significant element in geriatric assessment. It evaluates the capacity of daily life activity performance through BADL and instrumental ADL (IADL) [6,7]. BADL assesses the daily needs of skills such as bathing, dressing, toileting, and feeding, commonly assessed by the Katz index of independence in ADL (Katz ADL) [8]. IADL assesses complex functional activities like shopping, using the telephone, transportation, and laundry, along with other elements evaluated through the Lawton-Brody IADL [4,7]. Some routine cultural and religious rituals involve complex functional activities. In the Islamic religion, five prayers per day require Wudu (ablution). Wudu is a religious ritual involving the complex actions of washing the face and extremities in a specified sequence before each prayer. Therefore, it can be considered an indicator of functional status in the Muslim community [9].

Primary healthcare centers (PHCCs), managed by family physicians, play a crucial role in comprehensive geriatric assessment, including functional status assessment, management, and prevention of chronic diseases, along with the potential identification of barriers including medical and sociodemographic factors [10].

It is essential to evaluate factors leading to functional decline that eventually can lead to dependency and low quality of life in elderly people. Multiple studies have reviewed specific factors such as body aches, fall history, cognitive and mental status, polypharmacy, and its relation to functional decline [4,11]. With an increasing geriatric population in Saudi Arabia, it is crucial to focus on geriatric care provision and study relevant conditions and problems that may influence their overall health and quality of life. In Saudi Arabia, there is a dearth of literature on geriatric assessment, especially related to functional capacity in this age group. It is vital to know the level of functional abilities of the geriatric population before designing suitable interventions to address health issues and improve their quality of life. In this context, the current study aims to assess the functional abilities of geriatric patients attending PHCCs in the Qassim Region and identify factors associated with functional capacity decline among these patients.

## Materials and methods

Study design and setting

The study was a cross-sectional survey conducted among geriatric patients aged 65 years and older. The participants were selected from PHCCs in Buraidah city, Qassim Region, Saudi Arabia. The survey was carried out from June to October 2023.

Sample size calculation

The sample size was calculated using OpenEpi statistical software (www.OpenEpi.com). According to the 2019 national statistics of the geriatric population, the estimated geriatric population aged 65 years and older in the Qassim Region was 46,846 people [12]. The reference criterion for functional impairment was taken from a study by Al-Qahtani (2020), which showed that approximately 72% of the study participants did not have functional impairment in BADL [4]. Based on a 95% confidence interval and a 5% margin of error, the calculated sample size was 310 people.

Sampling technique

A two-stage cluster sampling was done. In the first stage, five PHCCs in Buraidah, Qassim Region, were selected. In the second stage of sampling, 62 participants, including 31 males and 31 females, were recruited from each selected PHCC. All elderly patients who visited the selected PHCCs and met the inclusion criteria were requested to participate in the survey until the required sample size from the center was attained.

Selection criteria

Saudis aged 65 years and older of both genders who agreed to participate were included in the study, while patients who were in acute pain at the time of presentation or had cognitive decline or neuromuscular conditions such as Parkinson’s disease, stroke, or paralysis were excluded from the study.

Sociodemographic section

This section encompassed questions regarding age, gender, marital status, education, living companionship, household status, and financial support. It also gathered information about chronic illnesses, polypharmacy, and the history of falls.

Research instrument

An interviewer-administered, semi-structured questionnaire was designed for the survey. The questionnaire had four sections, including sociodemographic characteristics, the Katz ADL, the Wudu assessment, and the Lawton-Brody IADL.

Katz ADL

The functional status of BADL was assessed using the Katz ADL in the geriatric age group 65 years and older, which involved bathing, dressing, toileting, transferring, continence, and feeding. Scoring was obtained using two criteria: independence was scored as 1 point given for those who didn’t need supervision or any assistance in doing the above-mentioned activities, and a score of 0 indicated dependence, representing those who required substantial assistance and supervision to complete their daily activities [13]. A total score of 6 signifies a high level of independence, while a score of 0 signifies significant dependency. The Katz ADL was extensively used in assessing the functional status of BADL in the elderly population [14].

Wudu Assessment

Wudu (ablution) is a daily religious activity for Muslims. It involves systemically washing body parts, starting with the hands, face, mouth, nose, ears, head, and lower extremities, before each of the five daily prayers. The assessment of the Wudu performance included three categories: independent, partially dependent, or dependent, based on the level of assistance required. Individuals with partial dependence were able to perform Wudu with some assistance, while those with total dependence needed complete assistance from another person.

Lawton-Brody IADL

The Lawton-Brody IADL consists of the following activities: ability to use telephones, shopping, food preparation, housekeeping, laundry, transportation, medication responsibility, and handling finances. The scoring system consisted of ratings: 0 for dependence, 1 for partial dependence, and 2 for complete independence. In assessing the level of dependence (independent, partially dependent, dependent), scoring was adjusted to prevent gender bias, employing distinct criteria for female and male participants. For females, all items were taken into account. Thus, a score of 8 for females indicated high IADL and independence. However, while calculating scores for males, food preparation, housekeeping, and laundry activities were omitted. Therefore, a total score of 5 indicated independent functional status for males [15,16].

Data collection

The data was collected through direct interviews using an interviewer-administered questionnaire. Data was collected by family physicians who were trained for data collection for this project. We introduced and briefly explained the research study to the potential participants and gained informed consent. We used a Google Form (Google LLC, California, USA), which contained consent prior to starting the questionnaire.

Data analysis

The data from Google Sheets (Google LLC, California, USA) was transferred to Microsoft Excel (Microsoft Corporation, Washington, USA), where data coding was done. After coding and data cleaning, the data was transferred to SPSS Statistics version 21 (IBM Corp. Released 2012. IBM SPSS Statistics for Windows, Version 21.0. Armonk, NY: IBM Corp.) for analysis. Frequencies and percentages were used to describe categorical variables and the mean, median, and standard deviation for continuous variables. To determine the associations of sociodemographic characters with the Lawton-Brody IADL, a chi-square test was used, while the associations of sociodemographic characters with the Katz ADL scores were determined by employing the Mann-Whitney U test and Kruskal-Wallis test. Non-parametric tests were employed as the Katz ADL scores lacked normal distribution, as confirmed through the Shapiro-Wilk test for normality. The correlation between Wudu performance and the Katz ADL and Lawton-Brody IADL scores was determined by the Pearson correlation coefficient. A statistically significant result was defined as a p-value of <0.05.

Ethical considerations

Ethical approval was obtained from the Regional Research Ethics Committee of Qassim Region (approval number: 607-44-14249). Permission was obtained from the PHCC directors. Informed consent was obtained from all study participants, and the questionnaire was kept anonymous. Data was coded in the database using a unique identification number, and all data was maintained confidential.

## Results

The questionnaire was distributed to 357 potential participants attending PHCCs in Buraidah, Qassim Region. Out of these, 310 patients responded and completed the study questionnaire, leading to a response rate of 87%.

The age of the respondents ranged from 65 to 96 years, with a mean of 71.95 (±7.02) years. Table 1 shows the demographic characteristics of the participants. Male and female study participants had equal representation. Around three-fourths of the study participants (n=231, 74.5%) belonged to the 65-75-year-old age group. The majority of the respondents (77.7%) were married. A total of 131 (42.3%) study participants were non-schooled. A vast majority of the respondents (91.9%) had chronic disease, and about 60% were on polypharmacy.

**Table 1:** Sociodemographic characteristics of the study participants (n=310)

Sociodemographic characteristics		Number	Percent
Gender	Female	155	50%
	Male	155	50%
Age in years	65-75	231	74.5%
	76-85	63	20.3%
	>85	16	5.2%
Marital status	Married	241	77.7%
	Separated	4	1.3%
	Widowed	65	21.0%
Education	Diploma	28	9.0%
	Elementary	61	19.7%
	Non-schooled	131	42.3%
	Secondary	68	21.9%
	University	22	7.1%
Companionship	Living with children	25	8.1%
	Living in own house	285	91.9%
Household status	Owned	297	95.8%
	Rental	13	4.2%
Financial support	Benefits	59	19.0%
	Family	120	38.7%
	Pension	131	42.3%
Chronic diseases	Yes	285	91.9%
	No	25	8.1%
Polypharmacy	Yes	186	60%
	No	124	40%

**Figure 1:**
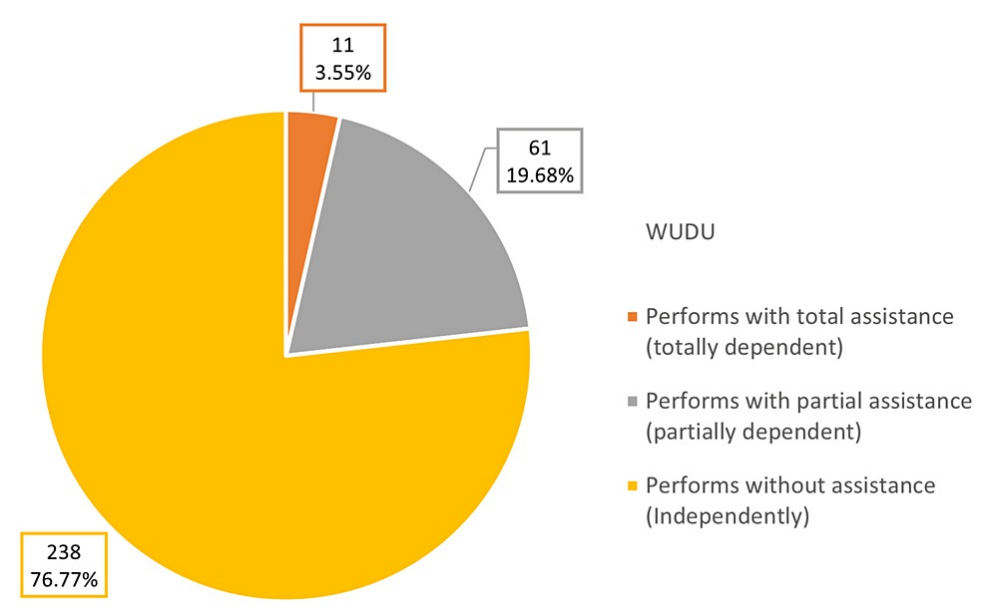
Functional capacity of performing Wudhu among geriatric patients attending PHCCs in Qassim Region (n=310) PHCCs: primary healthcare centers

The study participants were questioned about their history of falls. Among them, 46 females (14.84%) and 34 males (10.96%) reported a previous history of falls. Figure 1 displays the functional capacity of performing Wudu among the study participants. A total of 238 (76.77%) reported being able to perform Wudu without assistance, while 61 (19.68%) needed partial assistance.

Table 2 presents the assessment results based on the Katz ADL. The analysis indicates that a significant majority of respondents demonstrated independence in the ADL such as feeding (99.4%), transferring (95.5%), and dressing (93.9%). However, the ADL with the highest dependence rates included bathing (13.2%) and continence (11%).

**Table 2:** Katz ADL among geriatric patients attending PHCCs in Qassim Region (n=310) ADL: activities of daily living

Katz ADL		Number	Percent
Bathing	Dependence "Need help with bathing more than one part of the body, getting in or out of the tub or shower. Requires total bathing."	41	13.2%
	Independence "Bathes self completely or needs help in bathing only a single part of the body such as the back, genital area or disabled extremity."	269	86.8%
Dressing	Dependence "Needs help with dressing self or needs to be completely dressed."	19	6.1%
	Independence "Get clothes from closets and drawers and put on clothes and outer garments complete with fasteners. May have to help to tie shoes."	291	93.9%
Toileting	Dependence "Needs help transferring to the toilet, cleaning self or uses bedpan or commode."	28	9.0%
	Independence "Goes to the toilet, gets on and off, arranges clothes, cleans genital area without help."	282	91.0%
Transferring	Dependence "Needs help in moving from bed to chair or requires a complete transfer."	14	4.5%
	Independence "Moves in and out of bed or chair unassisted. Mechanical transfer aids are acceptable."	296	95.5%
Continence	Dependence "Is partially or totally incontinent of bowel or bladder."	34	11.0%
	Independence "Exercises complete self-control over urination and defecation."	276	89.0%
Feeding	Dependence "Needs partial or total help with feeding or requires parenteral feeding."	2	0.6%
	Independence "Gets food from a plate into the mouth without help. Preparation of food may be done by another person."	308	99.4%

Table 3 displays the Lawton-Brody IADL assessment results. Approximately 72.9% of participants exhibited the capability to independently use and operate a telephone. Notably, 170 (54.8%) participants expressed the need for assistance with their laundry-related activities. Moreover, a substantial 75.8% (235 respondents) demonstrated full responsibility for managing their medication, ensuring correct dosages, and adhering to scheduled times.

**Table 3:** Lawton-Brody IADL among geriatric patients attending PHCCs in Qassim Region (n=310) IADL: instrumental activities of daily living, PHCCs: primary healthcare centers

Lawton-Brody IADL		No.	%
Ability to use the telephone	Operates telephone on own initiative-look up and dial numbers, etc.	226	72.9%
	Dials a few well-known numbers	51	16.5%
	Answers telephone but does not dial	22	7.1%
	Does not use the telephone at all	11	3.5%
Shopping	Takes care of all shopping needs independently	170	54.8%
	Shops independently for small purchases	33	10.6%
	Needs to be accompanied on any shopping trip	80	25.8%
	Completely unable to shop	27	8.7%
Food preparation	Plans, prepares, and serves adequate meals independently	131	42.3%
	Prepares adequate meals if supplied with ingredients	53	17.1%
	Heats, serves, and prepares meals, or prepares meals, or prepares meals but does not maintain an adequate diet	39	12.6%
	Needs to have meals prepared and served	87	28.1%
Housekeeping	Maintains house alone or with occasional assistance (e.g., "heavy work domestic help")	106	34.2%
	Performs light daily tasks such as dishwashing, bed-making	35	11.3%
	Performs light daily tasks but cannot maintain an acceptable level of cleanliness	35	11.3%
	Needs help with all home maintenance tasks	46	14.8%
	Does not participate in any housekeeping tasks	88	28.4%
Laundry	Does personal laundry completely	111	35.8%
	Launders small items-rinses stockings, etc.	29	9.4%
	All laundry must be done by others	170	54.8%
Mode of transportation	Travels independently on public transportation or drives own car	130	41.9%
	Arranges own travel via taxi, but does not otherwise use public transportation	47	15.2%
	Travels on public transportation when accompanied by another	69	22.3%
	Travel limited to taxi or automobile with the assistance of another	52	16.8%
	Does not travel at all	12	3.9%
Responsibility for own medication	Is responsible for taking medication in correct dosages at the correct time	235	75.8%
	Takes responsibility if medication is prepared in advance in separate dosage	60	19.4%
	Is not capable of dispensing own medication	15	4.8%
Ability to handle finances	Manages financial matters independently (budgets, writes checks, pays rent, bills, goes to bank), collects and keeps track of income	196	63.2%
	Manages day-to-day purchases, but needs help with banking, major purchases, etc.	82	26.5%
	Incapable of handling money	32	10.3%

**Figure 2:**
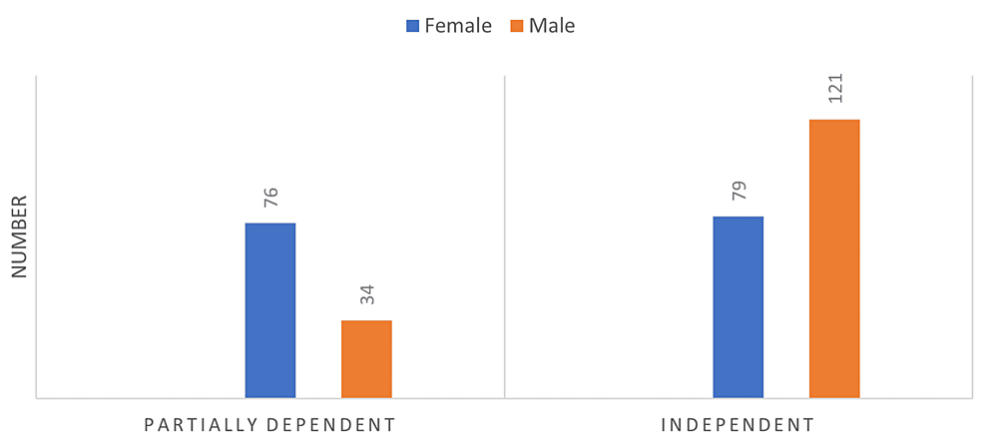
Lawton-Brody IADL scale among geriatric patients by gender attending PHCCs in Qassim Region (n=310) Independence in IADL categories based on a total score of 24 for females and 15 for males: dependent: score less than 8 for females and less than 5 for males, partially dependent: scores 8-17 for females and 5-11 for males, independent: total scores of 18-24 for females and 12-15 for males IADL: instrumental activities of daily living, PHCCs: primary healthcare centers

Figure 2 depicts the Lawton-Brody IADL for both genders. A total of 121 males and 79 females were capable of performing instrumental activities independently.

The overall mean Katz ADL score among study participants was 5.55 (±1.12), with a minimum of 0 and a maximum of 6. Table 4 shows the association between Katz ADL scores and the sociodemographic characteristics of the study participants. The functional capacity mean rank was higher for males (164.71) as compared to females (146.29). This difference was statistically significant at p=0.007. The respondents not on polypharmacy (p=0.002) and those without a history of falls (p<0.001) had statistically significantly better functional capacity as compared to their counterparts.

**Table 4:** Associations between the Katz ADL and sociodemographic data (n=310) ^¶^ Mann-Whitney U test, ^∞ ^Kruskal-Wallis test, ^* ^statistically significant at p<0.05

Sociodemographic variables		No. of respondents	Katz ADL (mean rank)	p-value
Gender^¶^	Female	155	146.29	0.007*
	Male	155	164.71	
Age in years^∞^	65-75	231	161.37	<0.001*
	76-85	63	149.87	
	>85	16	93	
Marital Status^∞^	Married	241	159.74	0.023*
	Separated	4	183.50	
	Widowed	65	138.04	
Education^∞^	Non-schooled	131	140.60	<0.001*
	Elementary	61	152.65	
	Secondary	68	173.23	
	Diploma	28	172.77	
	University	22	175.34	
Companionship^¶^	Living with children	25	126.44	0.012*
	Living own house	285	158.05	
Household status^¶^	Owned	297	155.85	0.622
	Rental	13	147.46	
Financial status^∞^	Benefits	59	146.89	0.008*
	Family	120	146.25	
	Pension	131	167.85	
Chronic diseases^¶^	No	25	177.40	0.057
	Yes	285	153.58	
Polypharmacy^¶^	No	124	168.37	0.002*
	Yes	186	146.92	
History of falls^¶^	No	230	167.92	<0.001*
	Yes	80	119.80	

On examining the associations between the Lawton-Brody IADL and sociodemographic data, gender (p<0.001), age (p<0.001), education (p<0.001), polypharmacy (p<0.001), and chronic disease (p=0.003) were significantly associated with the category of being independent (Table 5).

**Table 5:** Associations between Lawton-Brody IADL and sociodemographic data (n=310) ^¶^ Mann-Whitney U test, ^∞ ^Kruskal-Wallis test, ^*^ statistically significant at p<0.05

Sociodemographic variables		Lawton-Brody IADL			Statistical analysis		
		Partially dependent	Independent	Total	Statistical test	X2 value	p-value
Gender^¶^	Female	76	79	155	Pearson chi-square	24.856^a^	<0.001*
	Male	34	121	155			
Age^∞^	65-75	59	172	231	Pearson chi-square	41.586^a^	<0.001*
	76-85	38	25	63			
	>85	13	3	16			
Marital status^∞^	Married	65	176	241	Fisher-Freeman-Halton exact test	33.554^a^	<0.001*
	Separated	2	2	4			
	Widowed	43	22	65			
Education^∞^	Non-schooled	70	61	131	Pearson chi-square	56.250^a^	<0.001*
	Elementary	28	33	61			
	Secondary	10	58	68			
	Diploma	0	28	28			
	University	2	20	22			
Companionship^¶^	Living with children	17	8	25	Pearson chi-square	12.559^a^	<0.001*
	Living own house	93	192	285			
Household status^¶^	Owned	103	194	297	Fisher's exact test	-	0.234
	Rental	7	6	13			
Financial support^∞^	Benefits	29	30	59	Pearson chi-square	29.240^a^	<0.001*
	Family	57	63	120			
	Pension	24	107	131			
Chronic diseases^¶^	No	2	23	25	Pearson chi-square	8.972^a^	0.003*
	Yes	108	177	285			
Polypharmacy^¶^	No	28	96	124	Pearson chi-square	15.030^ a^	<0.001*
	Yes	82	104	186			
History of falls^¶^	No	67	163	230	Pearson chi-square	15.715^a^	<0.001*
	Yes	43	37	80			

A Pearson correlation coefficient was computed to assess the association between the Katz ADL and Lawton-Brody IADL. There was a positive correlation between the two variables (r=0.607, p<0.0001). The performance scores for Wudhu were also positively correlated with the Katz ADL (r=0.636, p<0.0001) and Lawton-Brody IADL (r=0.60, p<0.0001). In terms of ADL, individuals who performed Wudu without assistance (231 in total) demonstrated independence in bathing activities (p<0.001), mirroring a similar trend in dressing activities for those who independently performed Wudu.

## Discussion

The increase in the elderly population has drawn the attention of the healthcare system toward studying factors and effects that encourage the provision of high-quality healthcare [17]. Functional status is a significant element of comprehensive geriatric health assessment in the elderly population. It is important to study the impact of diseases and socioeconomic factors that can influence physical activity, mobility, and ADL function, leading to a decreased quality of life.

In this study, we assessed the functional status of the geriatric population attending PHCCs using the Katz ADL and Lawton-Brody IADL indices. We also studied the associations between sociodemographic characteristics, chronic diseases, fall history, and polypharmacy with the Katz ADL and Lawton-Brody IADL indices.

The mean age of our study participants was 71.95 (±7.02), with a majority age group range between 65 and 75 years. Comparable findings were observed in a Brazilian study, where 187 females and 85 males participated, with a mean age of 73.1 (±8.3) and 71.1 (±8.1) years, respectively [11]. In another study in Khamis Mushait, Saudi Arabia, conducted on 385 males, the participants’ mean age was 70.6 years, with a range between 60 and 69 years [4]. Another recent study by Al Anazi had 203 respondents with a mean age of 72.3 years. Over half (52.71%) of the participants were female, aged 65 years and older, extending beyond 75 years [14].

In our study, a high proportion (91.9%) of the participants had chronic disease. This finding is in agreement with other studies conducted among geriatric populations, which showed a high prevalence of chronic diseases. A study conducted in Poland showed the prevalence of chronic disease as 57.98% of the total study population [18], while another study conducted in Portugal stated that 81.7% of geriatric patients suffer from chronic diseases [10]. The high prevalence of chronic diseases within our study population might also be attributed to the setting: a primary healthcare environment where most participants visited for ongoing care for their health conditions.

In our study, 25.8% of the participants reported a history of falls within three months or more. In a study from Poland, 30.28% reported falls over the past year [18]. Another study noted a 21.3% occurrence of falls among participants [4]. Therefore, the incidence of falls observed in our study population aligns with the incidence reported in other studies.

Our study was conducted in a religious Muslim community where a daily ritual is performing Wudu (ablution) prior to prayers. Wudu is a complex cleaning activity that involves systematically washing specific body parts. Therefore, we also assessed the degree of dependence on performing Wudu, categorized by assistance levels. In our study, 76.77% could perform Wudu independently, 19.68% required partial assistance (like using a chair or a pre-set setup), and 3.55% needed full assistance. Few studies evaluate Wudu independence in older individuals; only one, from 1997 in Saudi Arabia, found that 6% had impairment in performing Wudu [9].

The Katz ADL index in our study revealed high independence in feeding (99.4%), transferring (95.5%), and dressing (93.9%). Comparable findings in a Brazilian study highlighted feeding (95.6%), toileting (94.9%), and transferring (93.4%) as the most independent ADL, while bathing and continence posed the greatest dependence [19,20]. Similarly, our study revealed the same patterns, with bathing (13%) and continence (11%) being the most dependent ADL. Bathing is consistently reported as the least independent activity across multiple studies [9,11,20]. Additionally, a study in Khamis Mushait, Saudi Arabia, reported partial dependence in bathing (14.6%) and dressing (14%) among participants [4].

In our study, the Lawton-Brody IADL index assessment revealed notable independence in managing one's medications (75.8%), using the telephone (72.9%), and handling finances (63.2%). A majority of our participants were either managing chronic conditions or seeking healthcare for other issues. Ensuring independent medication management was achieved through a review of their medication routines. Consistent with our findings, a study involving 272 participants demonstrated similar independence in using the telephone (88.2%) and managing medication (82.7%) without assistance [11]. However, in our study, total dependence among participants in the Lawton-Brody IADL included laundry (54.8%), housekeeping (28.4%), and food preparation (28.1%). Comparable studies also reported total dependence on IADL, such as tidying the house (41.5%) and meal preparation (32.2%) [4,9]. Additionally, a study conducted in Qassim Region, Saudi Arabia, found dependence on IADL like shopping (55.6%) and financial management (57.5%) [9].

We studied the association between sociodemographic factors and the Katz ADL and Lawton-Brody IADL indices to explore their impact on the functional capacity of the geriatric population. In our study, the male participants were better in both ADL (p=0.007) and IADL (p<0.001) than the female participants. In another study, the female participants showed greater dependence on ADL, while in IADL, male participants were more independent than female participants, and the difference was statistically significant (p<0.001) [10]. In the current study, the participants aged between 65 and 75 years had the highest independence level in the Katz ADL and Lawton-Brody IADL, whereas those aged 85 years and older showed declined performance in ADL and IADL. This can be attributed to the aging process, having multiple chronic diseases, and frailty [10,14,21]. Other studies have also reported functional decline associated with the age of 85 years or more [10,14,18]. In our study, educated, married participants and those residing with a spouse in their own homes exhibited higher levels of independence in ADL and IADL. Similar findings were reported in a recent study [19].

Polypharmacy has become a common problem in clinical practice. It has shown significant issues in the geriatric population's physical health, mental status, and quality of life [18,22]. Earlier research has documented a rise in functional impairment in ADL and IADL associated with the use of four or more medications [4,10,18]. Our study showed similar findings. In our study, approximately 60% of participants were engaged in polypharmacy, with 82 (26.5%) participants demonstrating partial dependence on the Lawton-Brody IADL index (p<0.001). We studied the association between the history of falls using the Katz ADL and Lawton-Brody IADL indices. Among the 230 participants without a fall history, 163 were found to be independent in the IADL. Conversely, among the 80 individuals with a positive fall history within the same timeframe, 37 were independent (p<0.001). Notably, a separate study from Poland indicated an elevated risk of experiencing at least one limitation in ADL and IADL with an increased number of falls in the preceding year [18].

In the current study, the performance scores for Wudu exhibited positive correlations with the Katz ADL (r=0.636, p<0.0001) and Lawton-Brody IADL (r=0.60, p<0.0001) indices. Regarding ADL, individuals performing Wudu without assistance exhibited independence in bathing and dressing activities. These statistically significant associations underscore the potential value of Wudu as a clinical tool for gaining insight into the functional status of the elderly in Muslim communities.

Our study was interviewer-administered and provided a comprehensive assessment of the functional capacity of the study population using validated instruments. However, there are certain limitations to our study. It was a cross-sectional study design; therefore, temporal associations cannot be determined. For example, it cannot be established whether a history of falls resulted in reduced functional capacity or whether the reduction in functional capacity was preceded by the occurrence of falls among the study participants. Furthermore, our study was conducted in a single city, limiting its generalizability. Another limitation of the study was its reliance on self-reported data, which may introduce reporting bias when assessing the functional capacity of patients.

## Conclusions

Our study focused on the geriatric population, with the majority having chronic diseases. More than one-third of the study participants were on polypharmacy. The Katz ADL showed high independence in feeding, transferring, and dressing, while the Lawton-Brody IADL showed independence in medication and phone use. Gender, age, education, polypharmacy, and chronic disease had a statistically significant association with the Lawton-Brody IADL categories. Wudu performance had a positive correlation with both the Katz ADL and Lawton-Brody IADL indices. Further research is required to assess Wudu as a clinical tool for evaluating the functional status of the elderly in Muslim communities. Moreover, it is recommended to research polypharmacy to identify interventions aimed at decreasing its prevalence. We suggest that healthcare practitioners, particularly family physicians in PHCCs, provide health education to the geriatric population. As a history of falls was found to be associated with reduced functional capacity, it is crucial to implement strategies to prevent falls among the elderly population and to enhance the well-being of the geriatric population.
